# Effect of Drying Methods on Aroma Profiling of Large-Leaf Green Tea (*Camellia sinensis* var. Assamica) Determined by HS-SPME-GC-MS

**DOI:** 10.3390/foods14071275

**Published:** 2025-04-05

**Authors:** Zhengfei Luo, Linlong Ma, Yangtao Zhang, Yanhong Liu, Rui Yang, Xuean Dai, Tiantian Wang, Changmi Lv, Lifeng Zuo, Yanli Liu, Dan Cao, Haibo Yuan, Longfeng Yu, Xiaofang Jin

**Affiliations:** 1 Yunnan Key Laboratory of Tea Germplasm Conservation and Utilization in the Lancang River Basin, College of Biotechnology and Engineering, West Yunnan University, Lincang 677000, China; luophy@126.com (Z.L.); 15125951384@163.com (Y.L.); 15887891889@163.com (R.Y.); lzf13759358903@163.com (X.D.); 19527071516@163.com (T.W.); 17874128563@163.com (C.L.); m19869498400@163.com (L.Z.); 2 Key Laboratory of Tea Resources Comprehensive Utilization of Ministry of Agriculture and Rural Affairs, Fruit and Tea Research Institute, Hubei Academy of Agricultural Sciences, Wuhan 430064, China; malinlong@hbaas.com (L.M.); zkslyl@163.com (Y.L.); skyiswide@163.com (D.C.); 3 Lincang Inspection Testing and Certification Institute, Lincang 677000, China; 13988383009@163.com; 4 Tea Research Institute, Chinese Academy of Agricultural Sciences, Hangzhou 310008, China; 192168092@tricaas.com

**Keywords:** large-leaf tea variety, green tea, drying methods, volatile components

## Abstract

Drying methods play a crucial role in the formation of green tea aromas. This study investigated the aroma characteristics and volatile component profiles of large-leaf green tea under hot-air drying, pan-fired drying, and sun drying. The results revealed significant differences in the sensory aroma characteristics and volatile components of the large-leaf green tea among the three drying methods. The pan-fire-dried green tea (PDGT) exhibited a distinct roasted aroma, while the hot-air-dried green tea (HDGT) and sun-dried green tea (SDGT) displayed a faint scent and lasting aroma characteristics, with the SDGT additionally featuring a noticeable sun-dried odor. A total of 48 differential volatile components were identified, among which β-Ionone, (E)-β-Ionone, 2,2,6-Trimethylcyclohexanone, Dihydroactinidiolide, BenzeneacetAldehyde, 2-Pentylfuran, 1,1,6-Trimethyl-1,2-dihydronaphthalene, δ-Cadinene, β-Myrcene, Geranylacetone, o-Cymene, 6-Methyl-5-hepten-2-one, (E)-β-Ocimene, and BenzAldehyde were identified as the primary contributors to the aroma differences among the three large-leaf green teas. Additionally, 43 differential volatile compounds were found to be significantly correlated with at least one of the aroma types (floral, sweet, green, faint scent, nutty, or roasted). The findings of this study provide a theoretical foundation for understanding the formation of aroma qualities in large-leaf green tea and offer valuable insights for improving its aromatic characteristics.

## 1. Introduction

Green tea is the most produced and consumed type of tea in China, and is highly favored by consumers for its unique flavor qualities and significant health benefits [1,2]. Aroma as a key factor for evaluating the quality of green tea, greatly influences the selection and acceptance of tea by tea drinkers [2,3]. Green tea is made from fresh tea leaves through five key processes: spreading, fixation, rolling, and drying [4]. Among them, drying, as the final processing step, plays a crucial role in the formation and fixation of the tea’s aroma quality [5]. Under high temperatures, low-boiling-point, grassy-smelling alcohols and aldehydes volatilize significantly, while higher-boiling-point alcohols, ketones, and acids are retained better, thereby altering the content and relative proportions of aroma components [6,7]. Simultaneously, high temperatures enhance the oxidative degradation, isomerization, and Maillard reactions in the substances within the tea, ultimately forming an extremely complex yet harmonious green tea aroma [4,8].

Different tea plant varieties exhibit significant differences in the content and composition of aroma precursors, as well as the characteristics of endogenous glycosidase enzymes. Even when processed using the same techniques, the resulting tea aromas can vary markedly [9]. The large-leaf tea variety (*C. sinensis* var. assamica) is widely cultivated in the southwestern and southern regions of China. Known for its rich content of intrinsic compounds, it produces teas with distinct qualities compared to those made from *C. sinensis* var. sinensis [10,11]. Processing green tea from large-leaf tea varieties primarily employs three traditional drying methods: hot-air drying, pan-fired drying, and sun drying [12]. Among them, sun drying is more commonly applied to large-leaf tea varieties than to *C. sinensis* var. sinensis [13]. For example, sun-dried green tea produced from the Yunnan large-leaf tea variety is particularly renowned in Yunnan. It can be drunk directly or used as a raw material for processing Pu-erh tea [14,15].

Different drying methods significantly alter the aroma of green tea. For example, pan-fired drying produces a richer array of volatile compounds compared to hot-air drying and is conducive to the formation of chestnut-like and roasted aromas in green tea [16]. During sun drying, the extensive degradation of fatty acids and carotenoids into aldehydes and terpenoid ketones contributes to the faint scent characteristic of sun- dried green tea [17]. However, previous research has primarily focused on *C. sinensis* var. sinensis, with most attention given to pan-fired drying, hot-air drying, and novel drying techniques [16,18]. There has been no systematic study on the aromatic qualities of large-leaf green tea under different drying methods. Therefore, this study utilized fresh leaves of the Mengku large-leaf tea variety (one bud and two leaves) as material. After spreading, fixation, and rolling, the leaves were processed using three drying methods: hot-air drying, pan-fired drying, and sun drying, resulting in hot-air drying green tea (HDGT), pan-fired drying green tea (PDGT), and sun-drying green tea (SDGT). The volatile components of these three large-leaf green teas were systematically analyzed using headspace solid-phase microextraction combined with gas chromatography–mass spectrometry (HS-SPME-GC-MS) and sensory evaluation methods, and key aroma-active components were identified. The aim of the research was to understand the aroma qualities of large-leaf green tea under different drying methods, and provide a theoretical basis for enhancing its aroma quality.

## 2. Materials and Methods

### 2.1. Experimental Materials

In April 2023, fresh leaves (FL) of the Mengku large-leaf variety (one bud and two leaves) were harvested at the experimental base of West Yunnan University (located in Mengku Town, Shuangjiang County). The FL underwent spreading, fixation, and rolling, followed by processing into green tea using three drying methods: hot-air drying, pan-fired drying, and sun drying. Specifically, the spreading process involved placing fresh leaves at a density of 0.75 kg/m^2^ under conditions of room temperature 18 °C~20 °C, and approximately 75% relative humidity until the moisture content in the leaves decreased to around 65%. For fixation, the spread leaves were processed in a drum fixation machine (6CST-80, Hangzhou Chunjiang Tea Machinery Co., Ltd., Zhejiang, China) at 280 °C for 2 min. Rolling was carried out using a rolling machine (6CR-65, Zhejiang Chunjiang Tea Machinery Co., Ltd., Hangzhou, China) for 30 min. For drying, HDGT was dried in a hot-air dryer (6CHM-901, Zhejiang Fuyang Tea Machinery Co., Ltd., Hangzhou, China) at 110 °C~120 °C for 10~15 min, followed by 80 °C for 15~20 min. PDGT was processed in a drum fixation machine (6CST-80, Zhejiang Chunjiang Tea Machinery Co., Ltd., Hangzhou, China) at 180 °C~250 °C for 25 min, followed by 90 °C~95 °C for 30 min. SDGT was dried in bamboo trays under sunlight at 25 °C~30 °C, with the leaves turned every hour. The moisture content of all tea samples was controlled to below 6%, and the moisture content of tea samples using a rapid moisture analyzer (HS153, Mettler Toledo, Zurich, Switzerland). Finally, 500 g samples of FL, HDGT, PDGT, and SDGT were collected, each in triplicate, and stored in a −80 °C freezer for further use.

### 2.2. Experiment Methods

#### 2.2.1. Sensory Evaluation and Quantitative Descriptive Analysis (QDA)

The aroma characteristics of the tea samples were subjected to sensory evaluation according to GB/T 23776-2018 [19]. Meanwhile, six experienced evaluators (three females and three males) conducted QDA of the tea samples’ aromas, using the method developed by Yue et al. (2023) [20]. In brief, precisely 3.0 g of each tea sample was weighed and brewed with 150 mL of boiling water for 5 min. The tea infusion was then transferred into a black-stoppered conical flask and randomly coded. After assessing the aroma characteristics of all tea samples, six primary aroma types (green, floral, sweet, nutty, roasted, and faint scent) were selected for QDA. A 10-point scale was used to rate the intensity of these six aroma attributes in the tea infusion, where 0 = absent, 3 = weak, 5 = moderate, 7 = strong, and 10 = very strong.

#### 2.2.2. Determination of Volatile Components Content by HS-SPME-GC-MS

The tea samples were retrieved from the −80 °C freezer and subjected to freeze-drying. They were then ground using liquid nitrogen and vortex mixed to ensure homogeneity. Precisely 0.5 g of each sample was weighed into a headspace vial, followed by the addition of 5 mL of saturated NaCl solution and 20 μL of internal standard solution (10 μg·mL^−1^ 3-Hexanone-2,2,4,4-d4). The mixture was shaken at a constant temperature of 60 °C for 5 min. A 120 µm DVB/CWR/PDMS fiber was inserted into the headspace vial for HS-SPME for 15 min, followed by desorption at 250 °C for 5 min. The extracted compounds were then separated and identified using GC-MS. GC–MS analysis was carried out using a GC system (8890, Agilent Technologies, Santa Clara, CA, USA) coupled with an MS detector (7000D, Agilent Technologies, Santa Clara, CA, USA). Chromatographic conditions: A DB-5MS capillary column (30 m × 0.25 mm × 0.25 μm, Agilent J&W Scientific, Folsom, CA, USA) was used, with high-purity helium (purity ≥ 99.99%) as the carrier gas at a flow rate of 1.2 mL/min. The injector temperature was set to 250 °C, and the injection was performed in splitless mode with a solvent delay of 3.5 min. The temperature program was as follows: hold at 40 °C for 3.5 min, increase to 100 °C at a rate of 10 °C/min, then to 180 °C at 7 °C/min, and finally to 280 °C at 25 °C/min, holding for 5 min. Mass spectrometry conditions: An electron ionization source was used, with the ion source temperature set to 230 °C, the quadrupole temperature to 150 °C, and the MS interface temperature to 280 °C. The electron energy was 70 eV, and the scanning mode was selective ion monitoring, with precise scanning of qualitative and quantitative ions.

#### 2.2.3. Quantification of Volatile Components and Odor Activity Values (OAVs)

For each component, one quantitative ion and two or three qualitative ions were selected. In the mass spectra of the samples after background subtraction, the presence of both the selected qualitative and quantitative ions served as the identification criterion. The quantitative ion was used for integration and calibration to enhance the accuracy of quantification. The relative content of volatile component *i* (X*_i_*) was calculated using the following formula:

(1) $$X_{i}=\frac{V_{s}\times C_{s}\times I_{i}}{1000\times M\times I_{s}}$$

In this equation, vs. is the volume of the internal standard (μL), C*_s_* is the concentration of the internal standard (μg·mL^−1^), M is the mass of the sample (g), I*_s_* is the peak area of the internal standard, and I*_i_* is the peak area of component *i* in the sample.

The OAV of each volatile component *i* (OAV*_i_*) in the sample was calculated using the following formula:

(2) $${\mathrm{OAV}}_{i}=\frac{X_{i}}{T_{i}}$$

In this equation, X*_i_* is the concentration of component *i* (μg·g^−1^), and T*_i_* is the odor threshold of compound component *i* (μg·g^−1^).

#### 2.2.4. Data Analysis

All experiments were conducted with three biological replicates. Bar graphs were generated using GraphPad Prism 5 software. Principal component analysis (PCA) was performed using SIMCA 14.1 software. Differential analysis was conducted using SPSS 19.0 software. Radar charts, heatmaps, Venn diagrams, and correlation analyses were created using an online platform available at https://www.chiplot.online (accessed on 12 February 2025).

## 3. Results and Discussion

### 3.1. Sensory Evaluation of Aroma in Large-Leaf Green Tea Under Different Drying Methods

**Figure 1:**
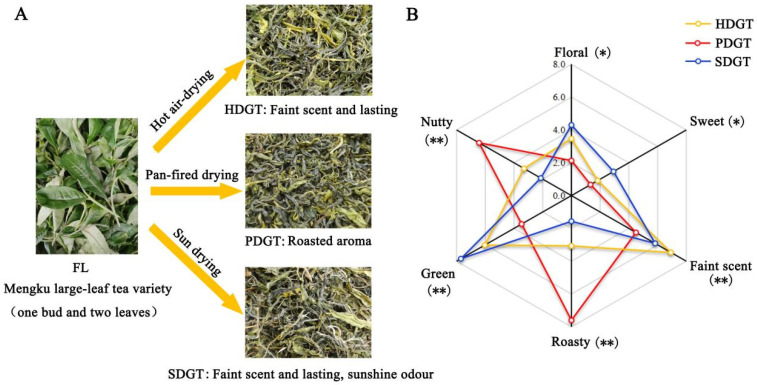
Sensory evaluation of aroma in large-leaf green tea under different drying methods. (**A**) Sensory description of aroma. (**B**) QDA radar chart. “*” indicates significant differences (*p* < 0.05), “**” indicates highly significant differences (*p* < 0.01).

As shown in Figure 1A, significant differences were observed in the sensory aroma characteristics of large-leaf green tea processed by the three different drying methods. PDGT exhibited a pronounced roasted aroma, while HDGT and SDGT displayed faint scents and lasting aroma characteristics, with SDGT additionally featuring a distinct sun-dried odor. The aroma profiles of these large-leaf green teas prepared under different drying methods are consistent with previous findings in *C. sinensis* var. Sinensis [16,21]. To provide a more detailed and accurate description of the aroma characteristics of the three types of large-leaf green tea, six aroma attributes were evaluated by QDA. As shown in Figure 1B, significant differences were observed in all six aroma attributes among the three tea samples, with nutty, green, roasted, and faint scents showing highly significant differences (*p* < 0.01). PDGT exhibited the most intense roasted and nutty aromas, while the other four attributes were the weakest. HDGT scored the highest in faint scent, with the other five attributes falling between the values of PDGT and SDGT. SDGT demonstrated the most intense green, floral, and sweet aromas, along with the weakest roasted and nutty aromas. These results indicate that different drying methods uniquely influence the sensory characteristics and intensity of aromas in large-leaf green tea. These effects may stem from changes in the content of certain volatile compounds under different drying conditions, leading to distinct aromatic profiles [22].

### 3.2. Analysis of Volatile Components Profile in Large-Leaf Green Tea Under Different Drying Methods

**Figure 2:**
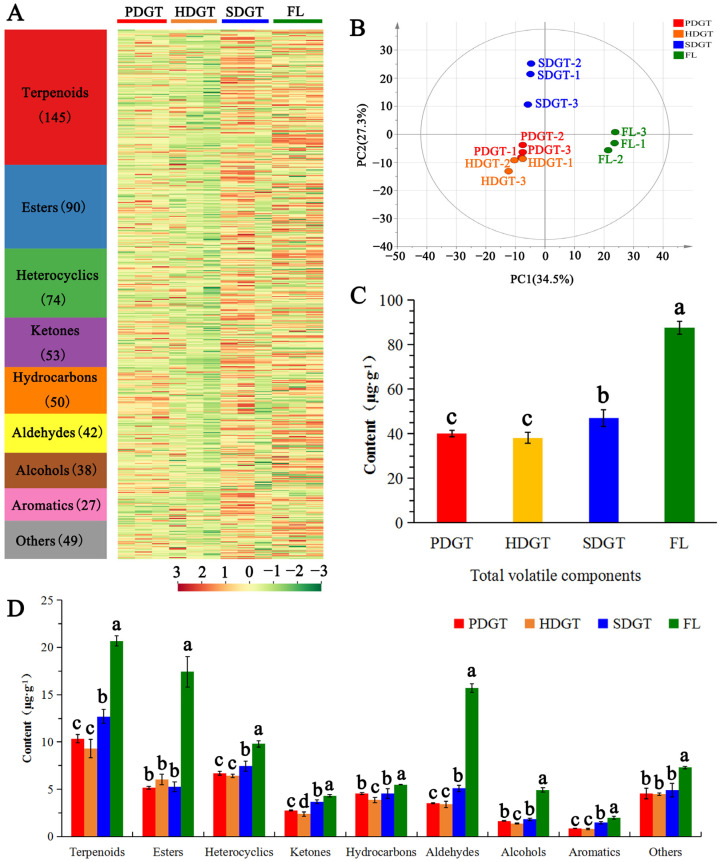
Analysis of volatile components in large-leaf green tea under different drying methods. (**A**) Heat map of the content of volatile components; (**B**) PCA score plot derived from the concentrations of volatile components; (**C**) the total content of volatile components; (**D**) the content of volatile component categories. Different lowercase letters indicate significant differences (*p* < 0.05).

The volatile components of PDGT, HDGT, SDGT, and FL were analyzed and identified using HS-SPME-GC-MS (Figure 2A). A total of 568 volatile components were detected, including 145 terpenoids, 90 esters, 74 heterocyclics, 53 ketones, 50 hydrocarbons, 42 aldehydes, 38 alcohols, 27 aromatics, and 49 others. The content of volatile components varied significantly among the four tea samples, with FL and SDGT exhibiting notably higher levels compared to PDGT and HDGT. PCA showed that FL was clearly distinguished from PDGT, HDGT, and SDGT, indicating that the drying process significantly altered the aroma components of FL when processed into green tea (Figure 2B). Meanwhile, PDGT and HDGT were distinctly separated from SDGT but clustered closer together, suggesting a certain similarity in their volatile components, while both differed significantly from SDGT. These results showed that different drying methods significantly influence the volatile component content of large-leaf green tea. The effect of sun drying on the volatile components of the green tea meant that it could be clearly distinguished from the samples that underwent hot-air drying and pan-fired drying.

As shown in Figure 2C, after FL is processed into PDGT, HDGT, and SDGT, the total content of volatile components significantly decreases by 54.25%, 56.52%, and 46.45%, respectively. The total volatile content of SDGT was 46.92 μg·g^−1^, significantly higher than that of PDGT and HDGT, while no significant difference was observed between PDGT and HDGT. The original volatile components in fresh tea leaves undergo substantial degradation and loss under high temperatures, leading to an overall decline in volatile content [6,7]. Meanwhile, similar results have also been found in other dried materials. For example, Zhang et al. [23] found that the operation of drying makes most of the volatile components in fresh *Malus asiatica* nakai decrease or vanish. Compared to SDGT, the higher drying temperatures of PDGT and HDGT resulted in significantly increased degradation and loss of volatile components, whereas these volatiles were better preserved in SDGT [6,7].

As indicated by the contents of different volatile component categories (Figure 2D), the significant degradation and loss of terpenoids, esters, and aldehydes in FL were key factors contributing to the lower total volatile content in PDGT, HDGT, and SDGT. Compared to FL, the contents of terpenoids in PDGT, HDGT, and SDGT decreases by 49.98%, 54.96%, and 38.58%, the content of esters decreases by 70.39%, 65.43%, and 69.76%, the content of aldehydes decreases by 77.52%, 78.24%, and 67.55%. These results exhibit substantial discrepancies when compared to the changes in aroma during the drying process of chili peppers. In chili peppers, the hot-air drying process led to an increase in acids, furans, and sulfide contents, while the levels of alcohols, esters, and olefins decreased [24]. Among the three drying methods, the contents of terpenoids, heterocyclics, aldehydes, and aromatics in the SDGT were significantly higher than those in the PDGT and HDGT, while no significant differences were observed between PDGT and HDGT. The contents of hydrocarbons and alcohols in PDGT and SDGT were significantly higher than those in HDGT, with no significant difference between PDGT and SDGT. Ketones showed significant differences among the three drying methods, with SDGT having the highest content at 3.68 μg·g^−1^, followed by PDGT at 2.76 μg·g^−1^, and HDGT with the lowest at 2.39 μg·g^−1^. No significant differences were observed in the contents of esters and others among the three drying methods.

### 3.3. Analysis of Differential Volatile Components in Large-Leaf Green Tea Under Different Drying Methods

**Figure 3:**
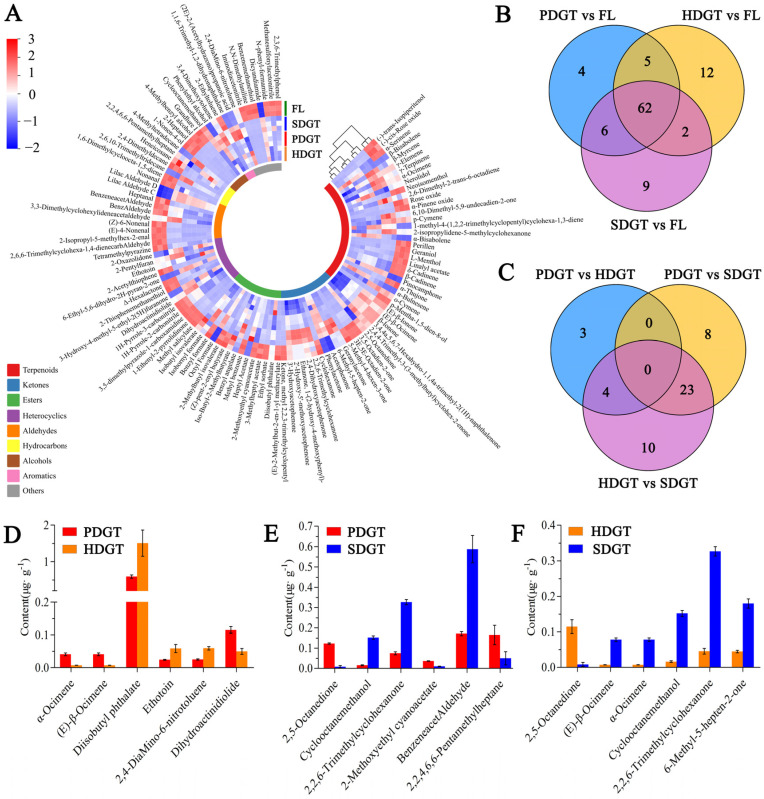
Analysis of differential volatile components in large-leaf green tea under different drying methods. (**A**) Heat map of the content of differential volatile components; (**B**) Venn diagram of the number of differential volatile components in the comparison groups PDGT vs. FL, HDGT vs. FL, and SDGT vs. FL; (**C**) Venn diagram of the number of differential volatile components in the comparisons between PDGT and HDGT, PDGT and SDGT, and HDGT and SDGT; (**D**) the content of the top six components with the largest fold changes in the comparison groups PDGT vs. HDGT; (**E**) The content of the top six components with the largest fold changes in the comparison groups PDGT vs. SDGT; (**F**) the content of the top six components with the largest fold changes in the comparison between HDGT and SDGT.

The OPLS-DA method was applied to differentiate and analyze the volatile components among PDGT, HDGT, SDGT, and FL. The screening of differential volatile components was based on a variable importance in the project (VIP) ≥ 1, a fold change ≥ 2 or fold change ≤ 0.5, and *p* < 0.05, and excluding volatile components with low contents (≤0.05 μg·g^−1^) across all four samples. As shown in Figure 3A, a total of 114 key differential volatile components were identified, including 32 terpenoids, 17 ketones, 16 esters, 14 heterocyclics, 11 aldehydes, 6 hydrocarbons, 6 alcohols, 3 aromatics, and 9 others. Most of these differential volatile components were significantly higher in FL compared to the three different drying methods of large-leaf green tea. The Venn diagram analysis revealed that the number of differential volatile components when comparing PDGT vs. FL, HDGT vs. FL, and SDGT vs. FL was 77, 81, and 79, respectively, while there were 4, 12, and 9, unique differential components, respectively (Figure 3B). Figure 3C shows that there were 48 differential volatile components among the three large-leaf green teas under the different drying methods. The number of differential volatile components when comparing PDGT vs. HDGT, PDGT vs. SDGT, and HDGT vs. SDGT was 7, 31, and 37, respectively, while the number of unique differential components was 3, 8, and 10, respectively. These findings indicate that the processing of fresh leaves into green tea results in significant changes in aroma components, and different drying methods influence the formation of the aroma in large-leaf green tea.

To further screen the markers in large-leaf green tea under different drying methods, the top six components with the largest fold changes in the comparisons between PDGT and HDGT, PDGT and SDGT, and HDGT and SDGT were selected for analysis based on their fold changes. In the PDGT vs. HDGT comparison, the six components with the largest fold changes were α-Ocimene, (E)-β-Ocimene, diisobutyl phthalate, ethotoin, 2,4-Diamino-6-nitrotoluene, and dihydroactinidiolide. Among these, α-Ocimene, (E)-β-Ocimene, and dihydroactinidiolide were present in higher amounts in PDGT compared to HDGT, while the others were lower in PDGT than in HDGT (Figure 3D). In the PDGT vs. SDGT comparison, the six components with the largest fold changes were 2,5-Octanedione, cyclooctanemethanol, 2,2,6-Trimethylcyclohexanone, 2-Methoxyethyl cyanoacetate, benzeneacetaldehyde, and 2,2,4,6,6-Pentamethylheptane. Among these, 2,5-Octanedione, 2-Methoxyethyl cyanoacetate, and 2,2,4,6,6-Pentamethylheptane were present in higher amounts in PDGT compared to SDGT, while the others were lower in PDGT than in SDGT (Figure 3E). In the HDGT vs. SDGT comparison, the six components were 2,5-Octanedione, (E)-β-Ocimene, α-Ocimene, cyclooctanemethanol, 2,2,6-Trimethylcyclohexanone, and 6-Methyl-5-hepten-2-one. Among these, only 2,5-Octanedione was present in higher amounts in HDGT compared to SDGT, while the others were lower in HDGT than in SDGT (Figure 3F).

### 3.4. Analysis of OAVs of Differential Volatile Components in Large-Leaf Green Tea Under Different Drying Methods

The OAV is a comprehensive indicator that considers both the concentration of volatile components and their odor thresholds, avoiding the misconception of solely relying on concentration to reflect their contribution to aroma. It is a method used to evaluate the importance of volatile components to the aroma quality of samples. In this study, the odor thresholds of the volatile components were primarily obtained from the existing literature [25,26,27]. From the 48 differential volatile components identified from the three different drying methods used for the large-leaf green tea, 14 volatile components with relatively high OAVs were selected, i.e., those with an OAV > 1 in at least one tea sample. As shown in Table 1, the mean OAVs of the 14 volatile components, in descending order, were β-Ionone, (E)-β-Ionone, 2,2,6-Trimethylcyclohexanone, Dihydroactinidiolide, Benzeneacetaldehyde, 2-Pentylfuran, 1,1,6-Trimethyl-1,2-dihydronaphthalene, δ-Cadinene, β-Myrcene, Geranylacetone, o-Cymene, 6-Methyl-5-hepten-2-one, (E)-β-Ocimene, and Benzaldehyde. Most of these components have been previously proven to be key contributors to tea aroma formation. For example, β-ionone is a key aroma component in Xinyang Maojian tea [28], and (E)-β-ionone is a key aroma component in Xiaokeng green tea [29]. Generally, substances with OAV > 1 are considered to contribute to aroma, while those with OAV > 10 are considered to make a significant contribution to aroma [25]. In this study, β-Ionone, (E)-β-Ionone, 2,2,6-Trimethylcyclohexanone, dihydroactinidiolide, benzeneacetaldehyde, 2-Pentylfuran, and 1,1,6-Trimethyl-1,2-dihydronaphthalene had OAVs greater than 10 in all three of the drying methods used for the large-leaf green tea. δ-Cadinene had OAVs greater than 10 in PDGT and HDGT, while β-Myrcene and Geranylacetone had OAVs greater than 10 in SDGT.

To further understand the differences in aroma formation in the large-leaf green tea prepared under different drying methods, the OAVs of 14 volatile components in PDGT, HDGT, and SDGT were compared and analyzed. In SDGT, the OAVs of all 14 volatile components, except for δ-Cadinene, were higher than those in PDGT and HDGT. Most of these substances have been confirmed to contribute to the formation of floral, sweet, and green aromas in tea. For example, (E)-β-Ionone is an important substance for the formation of floral aromas in the microwave second drying of green tea [30], β-Myrcene is a substance that helps enhance the sweet aroma of black tea [31], and Dihydroactinidiolide is a major contributor to the grassy and woody aromas in sun-dried green tea [21]. This may be the main reason why SDGT scored higher in sensory evaluations for floral, sweet, and green aroma types compared to PDGT and HDGT. Meanwhile, there is a significant antagonistic effect among volatile substances. The lower OAVs of these components in PDGT and HDGT may weaken the masking effects of faint scent, nutty, and roasted aroma types, ultimately making these aromas more perceptible [32,33]. Additionally, the OAVs of β-Ionone, (E)-β-Ionone, 2,2,6-Trimethylcyclohexanone, Dihydroactinidiolide, 2-Pentylfuran, δ-Cadinene, Geranylacetone, 6-Methyl-5-hepten-2-one, (E)-β-Ocimene, and BenzAldehyde in PDGT were higher than those in HDGT. Among these, 2-Pentylfuran, Geranylacetone, benzeneacetaldehyde, and (E)-β-Ionone have been confirmed to contribute to the formation of chestnut-like aromas in green tea [34,35]. This may be an important reason why PDGT scored higher in sensory evaluations for nutty and roasted aroma types compared to HDGT.

### 3.5. Correlation Analysis of Differential Volatile Components and Main Aroma Types

**Figure 4:**
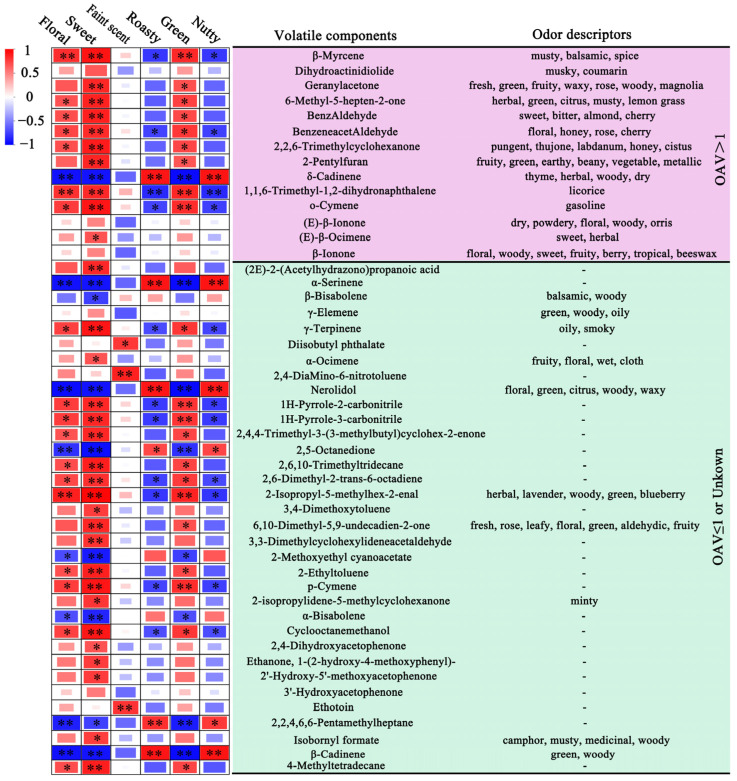
Correlation analysis of differential volatile components and main aroma types. Odor descriptors were obtained from reputable databases: The Good Scents Company (http://www.thegoodscentscompany.com), Perflavory Information System (http://perflavory.com (accessed on 10 December 2024)), The LRI & Odor Database—Odor Data (http://www.odour.org.uk/odour/index.html (accessed on 10 December 2024)), and Food Flavor Lab (http://foodflavorlab.cn/#/home (accessed on 10 December 2024)). “-” signifies that the odor descriptor was not found in the search. “*” indicates significant differences (*p* < 0.05), “**” indicates highly significant differences (*p* < 0.01).

To further understand the key volatile components contributing to the formation of the main aroma types in large-leaf green tea, a correlation analysis was conducted between the content of 48 differential volatile components and the sensory QDA scores of six aroma types in large-leaf green tea processed using three drying methods. As shown in Figure 4, a total of 43 differential volatile components were significantly positively or negatively correlated with at least one of the key six aroma types: floral, sweet, green, faint scent, nutty, and roasted. Among these, the majority of differential volatile components showed significant positive correlations with floral, sweet, and green aroma types, and their odor descriptions were consistent with the corresponding substances, further confirming the reliability of the study results. The low content of floral, sweet, and green aroma components reduced their masking effect on nutty and roasted aroma components (which showed no significant differences), resulting in a pronounced negative correlation between most differential components and nutty/roasted aroma characteristics [32,33].

As shown in Figure 4, the δ-Cadinene, α-Serinene, nerolidol, 2,5-Octanedione, 2-Methoxyethyl cyanoacetate, α-Bisabolene, 2,2,4,6,6-Pentamethylheptane, and β-Cadinene 8 differential volatile components showed significant negative correlations with floral, sweet, and green aroma types but positive correlations with nutty and roasted aroma types. Additionally, the correlation between differential volatile components and the faint scent aroma type of large-leaf green tea was not strong, showing significant positive correlations only with diisobutyl phthalate, 2,4-Dihydroxyacetophenone, and ethotoin, with correlation coefficients of 0.785, 0.874, and 0.800, respectively. Among the 14 volatile components with relatively high OAVs, β-Myrcene, 6-Methyl-5-hepten-2-one, benzAldehyde, benzeneacetAldehyde, 2,2,6-Trimethylcyclohexanone, 1,1,6-Trimethyl-1,2-dihydronaphthalene, and o-Cymene showed significant positive correlations with floral, sweet, and green aroma types. Among these, β-Myrcene, benzeneacetAldehyde, 1,1,6-Trimethyl-1,2-dihydronaphthalene, and o-Cymene showed significant negative correlations with nutty and roasty aroma types, with correlation coefficients all reaching 0.700. Furthermore, geranylacetone and 2-Pentylfuran, and (E)-β-Ocimene showed significant positive correlations with sweet aroma types, with correlation coefficients of 0.850, 0.857, and 0.674. Geranylacetone and 2-Pentylfuran showed a significant positive correlation with the green aroma type, with correlation coefficients of 0.688 and 0.669. Dihydroactinidiolide, (E)-β-Ionone, and β-Ionone showed no significant relationship with any of the six aroma types.

## 4. Conclusions

In this study, the differences in volatile components of large-leaf green tea prepared under three drying methods were systematically analyzed using HS-SPME-GC-MS combined with sensory evaluation. The results showed that the three drying methods significantly influenced the sensory aroma characteristics of large-leaf green tea. Pan-fire-dried green tea exhibited a distinct roasted aroma, while hot-air-dried and sun-dried green tea displayed faint and lasting aroma characteristics, with the sun-dried green tea additionally featuring a noticeable sun-dried odor. A total of 568 volatile components were detected in the large-leaf green tea prepared under the three drying methods, and 48 differential volatile components were identified. Among these, the effect of sun drying on the volatile components of green tea was clearly distinguished from that of hot-air drying and pan-fire drying. Fourteen volatile components with relatively high OAV were identified as the primary contributors to the aroma differences among the three types of tea. Forty-three differential volatile components were found to be significantly positively or negatively correlated with at least one of the aroma types (floral, sweet, green, faint scent, nutty, or roasted). Most of these differential volatile components showed positive correlations with floral, sweet, and green aromas, while exhibiting negative correlations with nutty and roasted aromas. This study provides a scientific analysis of the effects of three drying methods on the aroma of large-leaf green tea, offering a theoretical basis for improving its aroma quality.

## Figures and Tables

**Table 1:** Analysis of OAV of differential volatile components in Large-leaf green tea under different drying methods.

No.	Compounds	Threshold (ug·g^−1^) [21,22,23]	PDGT	HDGT	SDGT	Mean
1	β-Ionone	0.000007	133,873.74	77,360.89	162,112.84	124,449.16
2	(E)-β-Ionone	0.0002	4685.58	2707.63	5673.95	4355.72
3	2,2,6-Trimethylcyclohexanone	0.0001	745.71	454.17	3270.06	1489.98
4	Dihydroactinidiolide	0.0021	54.75	23.70	88.61	55.69
5	BenzeneacetAldehyde	0.0063	27.23	38.26	93.28	52.93
6	2-Pentylfuran	0.006	15.10	10.30	38.19	21.20
7	1,1,6-Trimethyl-1,2-dihydronaphthalene	0.0025	11.07	17.78	29.55	19.47
8	δ-Cadinene	0.0015	16.08	10.90	6.76	11.25
9	β-Myrcene	0.015	5.76	7.31	14.82	9.30
10	Geranylacetone	0.01	4.39	3.34	12.98	6.90
11	o-Cymene	0.01144	3.74	4.20	8.58	5.51
12	6-Methyl-5-hepten-2-one	0.05	1.11	0.89	3.60	1.87
13	(E)-β-Ocimene	0.034	1.20	0.21	2.30	1.24
14	BenzAldehyde	0.35	0.42	0.36	1.01	0.59

Note: PDGT represents pan-fire-dried green tea, HDGT represents hot-air-dried green tea, and SDGT represents sun-dried green tea.

## Data Availability

The original contributions presented in the study are included in the article. Further inquiries can be directed to the corresponding authors.
